# Potential Epidemic Vulnerability and Socioepidemiological Profile of SARS-CoV2 in the Brazilian Northeast Region

**DOI:** 10.3390/tropicalmed8040192

**Published:** 2023-03-27

**Authors:** Lohanna Valeska de Sousa Tavares, Antônio Júnior Alves Ribeiro, Denise Maria Christofolini

**Affiliations:** 1 Setor de Pós-Graduação do Centro, Universitário FMABC, Santo André 09060-590, SP, Brazil; 2 Instituto Federal de Educação, Ciência e Tecnologia do Ceará, Juazeiro do Norte 63047-040, CE, Brazil

**Keywords:** pandemics, SARS-CoV2, social vulnerability index, epidemiology, demography

## Abstract

Background: COVID-19 is a significant public health problem that can have a negative impact, especially in vulnerable regions. Objective: This study aimed to provide evidence that could positively influence coping with COVID-19 based on the relationship between the potential epidemic vulnerability index (PEVI) and socioepidemiological variables. This could be used as a decision-making tool for the planning of preventive initiatives in regions with relevant vulnerability indices for the spread of SARS-CoV-2. Methodology: We performed a cross-sectional study, with the analysis of the population characteristics of COVID-19 cases associated with neighborhoods’ PEVIs in the conurbation region of Crajubar, northeastern Brazil, through the mapping of socioeconomic–demographic factors and spatial autocorrelation. Results: The PEVI distribution indicated low vulnerability in areas with high real estate and commercial value; as communities moved away from these areas, the vulnerability levels increased. As for the number of cases, three of the five neighborhoods with a high–high autocorrelation, and some other neighborhoods showed a bivariate spatial correlation with a low–low PEVI but also high–low with indicators that make up the PEVI, representing areas that could be protected by public health measures to prevent increases in COVID-19 cases. Conclusions: The impact of the PEVI revealed areas that could be targeted by public policies to decrease the occurrence of COVID-19.

## 1. Introduction

COVID-19 is an acute infectious disease caused by a coronavirus identified as SARS-CoV-2 (severe acute respiratory syndrome, Coronavirus 2). This virus emerged in Wuhan, China, and quickly spread worldwide with a profound global impact, including increasing and recurrent rates of hospitalizations and mortality [1,2].

The COVID-19 pandemic, which was declared by the World Health Organization (WHO) on 11 March 2020, has led many countries to adopt unprecedented public health measures to contain its spread. As of 21 February 2023, 757,264,511 people worldwide had been infected with SARS-CoV-2, resulting in 6,850,594 deaths [3].

According to data released by the Ministry of Health, by 22 February 2023, Brazil had registered 36,967,328 accumulated cases and 698,381 deaths from SARS-CoV-2 [4]. From 26 February 2020 to 31 December 2022, the northeast region had an incidence of 12,561.4 cases/100 thousand inhabitants and a mortality of 233.3 deaths/100 thousand inhabitants, with the state of Paraíba experiencing the highest incidence (17,333 cases/100 thousand inhabitants), and Ceará the highest mortality (305.5 deaths/100 thousand inhabitants) [5].

However, the interior territories of Ceará quickly experienced an exponential proliferation of disease cases [6], especially in urban regions, such as the Metropolitan zone of Cariri, which covers 5456.01 km^2^ [7], and recorded 218,507 confirmed cases and 3537 deaths [6].

Through Complementary Law No. 78, this urban region was instituted in 2009. It included nine municipalities: Juazeiro do Norte, Crato, Barbalha, Jardim, Missão Velha, Caririaçu, Farias Brito, Nova Olinda, and Santana do Cariri [8].

**Figure 1:**
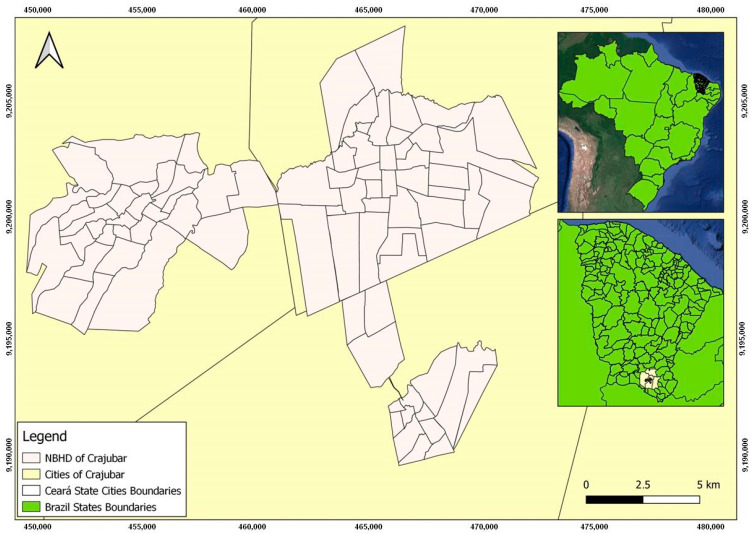
Geographic location and neighborhoods in the conurbation of the Crajubar region.

Figure 1 exhibits the cities of Crato, Juazeiro do Norte, and Barbalha, which have the three highest urbanization rates and represent a rich tourist heritage (religious, natural, historical, and events) as an economic growth pole, raising the promotion of the Crajubar urban agglomeration, where, specifically, they had accounted for more than 84,331 confirmed cases, with 1228 deaths reported, according to data released by the Health Department [8,9]. 

Crajubar’s high potential for economic development enshrines it as a large secondary urban center in the interior of Ceará, where most of the population is concentrated. However, advancing the growth of municipalities in this urban agglomeration has led to the growth of slums and the development of social problems, resulting in a poor quality of life in the community and rural exodus, as well as its consequences in public health, such as an increase in zoonoses and infectious diseases [10,11].

There is a need to critically evaluate epidemiological data regarding spatial relationships, which are crucial for understanding the dynamics of SARS-CoV-2 transmission at local, regional, and global scales. In this way, georeferencing is a helpful tool for the epidemiological surveillance of COVID-19 and other communicable diseases associated with social determinants in micro or macro-spatial analyses [2].

This study aims to provide evidence that can positively influence an individual’s ability to cope with COVID-19 through the relationship between the potential epidemic vulnerability index (PEVI) and socioepidemiological variables, which can be used as a decision-making tool given the planning of preventive initiatives for regions with relevant vulnerability indices for the spread of SARS-CoV-2.

Social vulnerability is defined as the degree to which a community is able to prepare for and respond to a natural or man-made disaster. The purpose of this index is to quantitatively assess social vulnerability at the census sector level and the cumulative incidence of reported COVID-19 cases in that area or region [12]; this can be optimized by adjusting the parameters of suitable scenarios and goals accordingly [13]. Marvel and others [14] presented a pandemic vulnerability index with geographic visualizations and a dashboard of county-level risk scores across certain US states. Another index model was designed around clinical factors that could determine the mortality and social factors and incorporate an infection rate to assess COVID-19 vaccination distributions [13].

A study [15] has shown with the reduction in vaccination rates for the most common diseases in children under 5 years of age in West Africa public health support is needed to ensure effective care during the COVID-19 pandemic, particularly in poor settings where preventive measures can save lives in the long term. As seen in rural areas of sub-Saharan West Africa, where tourism, fishing, and small traders represent the main income activities, the public health tools initially available to control disease, such as social distancing and isolation, generated socioeconomic impacts, with important consequences for health systems as well [16].

Historically, disadvantaged people have been highly vulnerable to emerging infectious diseases, especially when they emerge as a persistent epidemic [17,18]. According to study [19], vulnerabilities related to income inequalities and health infrastructure were the ones that most shaped the dynamics of the first wave of COVID-19 in Brazil from March to October 2020. Meanwhile, the second wave of the disease, which took place between November 2020 and June 2021, was influenced by the ideology and political orientation of municipalities that were focused on scientific denialism. In other countries, such as the US and China, low socioeconomic indicators were also associated with the distribution of COVID-19 incidence and lethality rates due to variations in personal hygiene, access to health care, and adherence to social distancing, and remote working recommendations [18,20].

Thus, it may be possible to provide evidence that can influence the confrontation of COVID-19 and other epidemics of infectious diseases in other parts of the world by assessing the potential of epidemic vulnerability, with the use of spatial and space-time grouping methods as a decision-making tool for the planning of intensive preventive actions. These findings could help public health services to better target intervention sites and undertake disease control, including targeting sites for early vaccination. This study represents an effort to contribute to existing geospatial research on COVID-19, but with a focus on under-resourced areas.

## 2. Materials and Methods

We undertook a cross-sectional study that analyzed the population characteristics of patients reported with COVID-19 and confirmed by molecular tests (RT-PCR or Antigen Test) or serological tests, which are associated with the construction of the PEVI of the Crajubar region. Crajubar is an urban agglomeration composed of three municipalities (Crato, Juazeiro do Norte, and Barbalha) in the interior of Ceará State, Brazil, which was composed through the mapping of socioeconomic and demographic factors raised by the Brazilian Institute of Geography and Statistics (IBGE) in the census from 2010.

Quantum Geographic Information System (QGIS) version 3.22.6, PostGre/PostGIS, EPI-INFO version 7.0, GeoDa version 0.9.9.10, and Excel were used to carry out the geographic correction procedures, statistical treatment, data processing, connection based on IBGE, spatial consultation, and the generation of maps.

### 2.1. Construction of the Potential Epidemic Vulnerability Index

This concept of social vulnerability has been widely used to understand how, despite its global distribution at local levels, COVID-19 has disproportionately affected some communities since the beginning of the pandemic. Thus, social vulnerability indices are an important tool to guide emergency responses and recovery efforts against disasters and infectious disease outbreaks. However, accurately quantifying social vulnerability remains a challenge, and the COVID-19 pandemic has highlighted a critical need to identify those most eligible and provide support to mitigate risks, such as understanding the relationship between social vulnerability and SARS-CoV-2 incidence. It is critical to understand the interplay between social determinants and implement risk mitigation guidelines that can reduce the spread of infectious diseases [21,22].

In this study, the variables for the construction of the PEVI were obtained from the 2010 IBGE Census, where the spatial database of Crajubar, which was acquired, could design in the SIRGAS 2000 reference system with a UTM Zone 24 South coordinate system and the alphanumeric database of the 2010 Brazilian census [7].

The geographic data (shapefile) provided by the IBGE had the census sector as the smallest aggregate geographic unit. However, the discretization of the data on the maps in this work took place through the neighborhoods of the municipalities of Crajubar since the calculation of the epidemiological data of COVID-19 was reported in this way.

Given the vast amount of information in the IBGE database, we sought to identify which variables most represented, from a collective point of view, the population’s vulnerability to the COVID-19 pandemic. Thus, several socioeconomic–demographic factors were tested based on cases from the 2010 Brazilian census by IBGE [7]. However, the seven factors most closely related to vulnerability toward COVID-19 were selected, as shown in Table 1.

The IBGE 2010 demographic census data, with all their variables in spreadsheets in the “.csv” format, were imported to the Postgre/PostGIS program. These seven variables were selected and subsequently connected to the image file for each neighborhood of Crajubar, with spatialized data necessary for the PEVI calculation.

Once the database was built, we analyzed its spatial integrity. Then, the indicators were standardized (normalization), which placed the data in the same unit through Equations (1) and (2) [23].

(1) $$I_{ps\left(P\right)}=\frac{I_{s}-I_{min}}{I_{max}-I_{min}}$$

(2) $$I_{ps\left(N\right)}=\frac{I_{s}-I_{max}}{I_{min}-I_{max}}$$

The variable *I_ps_* correspond to the standardized value of the indicator (*I_s_*) in the neighborhood (s). It is equivalent to the original value of the indicator (*I_s_*) in the neighborhood (s), and Imax and Imin are, respectively, the maximum and minimum values of the indicator (*I_s_*) within the universe of neighborhoods. Equation (1) was used for indicators that were positively associated with vulnerability (the lower the value of the indicator, the lower the vulnerability) and were called *I_ps_*_(*P*)_. As for indicators negatively associated with vulnerability (the lower the indicator, the greater the vulnerability), Equation (2), known as *I_ps_*_(*N*)_, was used.

Thus, the *PEVI_Crajubar_* was calculated from Equation (3) below, in which the *I_ps_* variable corresponds to the standardized value of the indicator (I) in the neighborhood, the *PEVI_Crajubar_* is equivalent to the *PEVI_Crajubar_* in the neighborhood, and the variable n corresponds to the total selected indicators. It is noteworthy that potential epidemic vulnerability to COVID-19 is higher for values closer to 1 and lower for values closer to 0.

(3) $$PEVI_{Crajubar}=\frac{\sum_{\;i=1}^{\;n}I_{ps}}{n}$$

This entire process, from the standardization of the indicators to the vulnerability classification of each neighborhood, was conducted in an Excel spreadsheet. The results were added to the QGIS 3.22.6 attribute table to generate the potential epidemic vulnerability to the COVID-19 map.

### 2.2. Regional Epidemiological Profile

Data on the epidemiological profile were obtained through the IntegraSUS platform in the State of Ceará. It is noteworthy that this state database contains all the cases that were tested for the disease since the first suspected case [9].

The socioepidemiological characteristics contained in the platform were taken from notification instruments such as those for private units, ESUS-VE epidemiological surveillance, the laboratory environment manager system (GAL) for confirmed cases, and the epidemiological surveillance coordination (Covep) for deaths. Such data depend on the notification of cases, which are nationally underestimated, alongside the completeness of filling in and the veracity of data in the forms.

The data, corresponding to the period between March 2020 and August 2022, were accessed and collected on 6 October 2022. Subsequently, we selected COVID-19 cases as confirmed by molecular or serological tests belonging only to the municipalities of Crato, Juazeiro do Norte, and Barbalha. Therefore, the inclusion criteria were the availability of information on the geographic location of the case and its laboratory confirmation.

Data were organized and reviewed in an Excel spreadsheet, excluding cases with incomplete or inconsistent information on epidemiological variables and correcting demographic data using the cartographic base of IBGE. It was necessary to guarantee the correct connection between spatial descriptive data and the information of the related cases, where each line in the spreadsheet with descriptive data had a corresponding spatial object (neighborhood) in the cartographic base (shapefile). Subsequently, the data were stored in the statistical program EPI-INFO, version 7.0, which allowed its tabulation to carry out the statistical analyzes of dispersion and central tendency by neighborhoods of the following variables: location, sex, age group, and race.

### 2.3. Spatial Analysis

The epidemiological data were added to the PEVI cartographic base in this phase. From this, it was possible to seek spatial relationships between the PEVI, the variables that compose it, and the number of COVID-19 cases per neighborhood.

We estimated the autocorrelation between the descriptive variables through the global Moran’s index (GMI). Moran’s spatial autocorrelation functions can reveal deep geographic information, perform dynamic spatial analysis, and lay the groundwork for the scale analysis of spatial locomotion [24,25]. The GMI is an inferential statistic; therefore, the analysis results are always interpreted within the context of its null hypothesis. Then, in the GMI statistic, the null hypothesis states that the attribute, having been analyzed, is randomly distributed among the cartographic features in the study area. In this study, the *p*-value that returned for all the tested data was statistically significant. Next, Moran’s local autocorrelation (IML) and spatial clusters were evaluated using the local indicators of spatial association (LISA) method alongside the GeoDa 0.9.9.10 software [26,27]. For the mensuration of spatial autocorrelation, generating a geographic/spatial proximity matrix was necessary.

Thus, IML corresponds to the local Moran index, which is calculated through Equation (4), where *Z_i_* is the difference between the attribute value at the location and the average of all attributes, *Z_j_* is the difference between the attribute value at the location and the average of all the attributes, and *W_ij_* refers to the weights or degrees of connectivity that are assigned according to the topological relationship between the *i* and *j* [28].

(4) $$I_{ML}=\frac{\sum W_{ij}Z_{i}Z_{j}}{\sum_{i=1}^{n}Z_{i}^{2}}$$

To this end, univariate cluster maps were constructed for the dependent variable (total number of COVID-19 cases in the neighborhoods) and the independent variables (PEVI and indicators in Table 1). In this analysis, the cartograms show the outline of four types of spatial clusters: high–high (regions formed by neighborhoods with high frequencies of the variable, surrounded by regions of high frequency); low–low (regions formed by neighborhoods with low frequencies of the variable, surrounded by regions of low frequency), high–low (regions formed by neighborhoods with high frequencies of the variable, surrounded by regions of low frequency), and low–high (regions formed by neighborhoods with low frequencies of the variable, surrounded by regions of high frequency) [26,27].

Likewise, the LISA method was used to conduct the bivariate analysis to assess the spatial correlation between the dependent and independent variables. In this case, we categorized five spatial cluster interpretations: non-significant (territories that did not form clusters, as their differences were not significant); high–high (regions formed by neighborhoods with high frequencies of the dependent variable and high frequencies of the independent variable); low–low (regions formed by neighborhoods with low frequencies of the dependent variable and low frequencies of the independent variable), high–low (regions formed by neighborhoods with high frequencies of the dependent variable and low frequencies of the independent variable) and low–high (regions formed by neighborhoods with low frequencies of the dependent variable and high frequencies of the independent variable) [26,27].

This study was approved by the research ethics committee of Centro Universitário Dr. Leão Sampaio (Report number 4,258,639).

However, this study had limitations in relation to sociodemographic data, as the latest available corresponded to the year 2010. Moreover, it was not possible to obtain data on mortality from the disease separated by neighborhood while impairing an analysis of the performance of severity. The use of data through the reporting system is also an important limitation since this is a precarious area of a developing country whose unequal socioeconomic distribution and access to health systems are related to underreporting. In addition, it was not possible to undertake a temporal evaluation corresponding to the peak waves of the cases experienced in the years studied and a correlation with vaccination, which also occurred disproportionately throughout the world and with the influences of scientific denialism due to the political polarization experienced by Brazil.

## 3. Results

**Figure 2:**
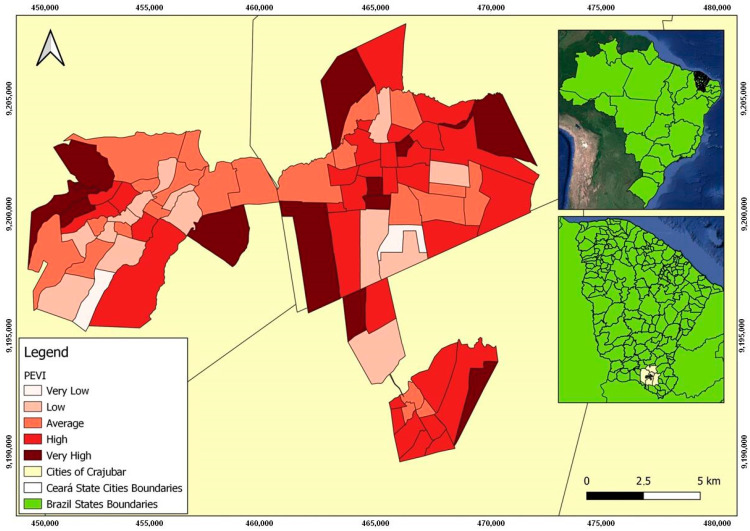
Distribution map of the Potential Epidemic Vulnerability Index in the urban area of Crajubar, northeastern Brazil.

The PEVI for the urban areas of Crajubar can be seen in Figure 2, which illustrates that the index was spatially well-stratified and retained a substantial similarity in the geographic distribution of the seven variables that compose it. When analyzing the PEVI distribution based on an empirical acknowledgment of the study area, it indicates a low vulnerability in regions with high real estate and commercial value, while vulnerability levels increase as neighborhoods move away from these areas.

**Figure 3:**
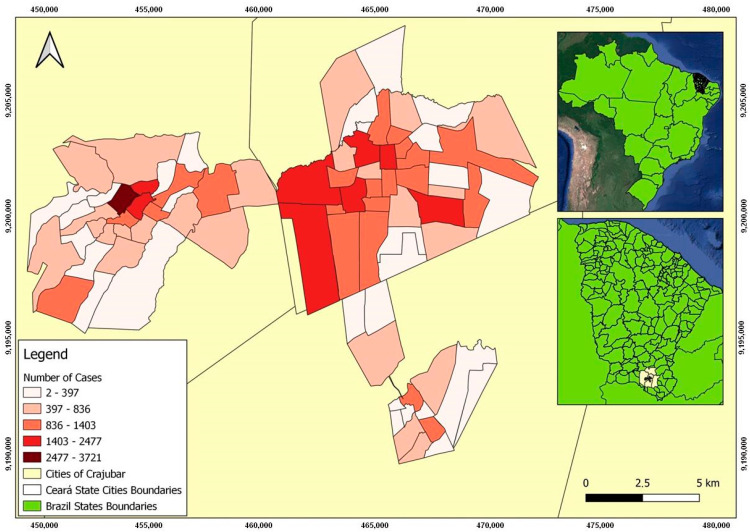
Distribution map of COVID-19 confirmed cases in the urban area of Crajubar, northeastern Brazil.

Between March 2020 and August 2022, Crato, Juazeiro do Norte, and Barbalha cities had 84,331 confirmed cases of COVID-19, 1228 deaths, and a lethality rate of 1.5%. After excluding rural areas and inconsistent data, 62,290 confirmed cases in Crajubar were identified and scrutinized, with spatial distribution as shown in Figure 3. Data on deaths by neighborhoods were unavailable on the digital platform, limiting its spatial analysis.

Of these cases, 73.7% were black and brown, 18.4% white, and 7.6% Asian. The indigenous race represented only 0.13% (82); the State of Ceará has 14 indigenous communities that are spread over 18 municipalities, with 568 self-identified Indians in the Crajubar region, according to the Institute of Research and Economic Strategy of Ceará (IPECE) [29].

Assessing the total cases by age showed that the median was 36 years, with the most affected group appearing between 20 and 39 years (45.3%). Children and adolescents from 0 to 19 years represented 13.5% of the cases, and older adults over 60 years represented 11.5%, with medians of 12 and 69 years of age, respectively. Females accounted for 35,515 (57%). The descriptive analysis is shown in Table 2.

According to the mean of the total cases (769), of the 81 analyzed neighborhoods, 21 had COVID-19 cases above 1000. Two neighborhoods in the city of Crato (Seminário and Vila Alta) reported the highest numbers, as shown in Figure 2. Both are among the neighborhoods with the highest population density in this municipality and have a high and medium PEVI, respectively, as shown in Figure 2.

However, it was noticed that the peripheral areas of Crajubar with high and very high PEVI had a low number of cases, which could be explained by underreporting, difficulties in accessing the health system, and diagnostic means.

Figure 3 illustrates the distribution map of COVID-19-confirmed cases in the analyzed region.

**Figure 4:**
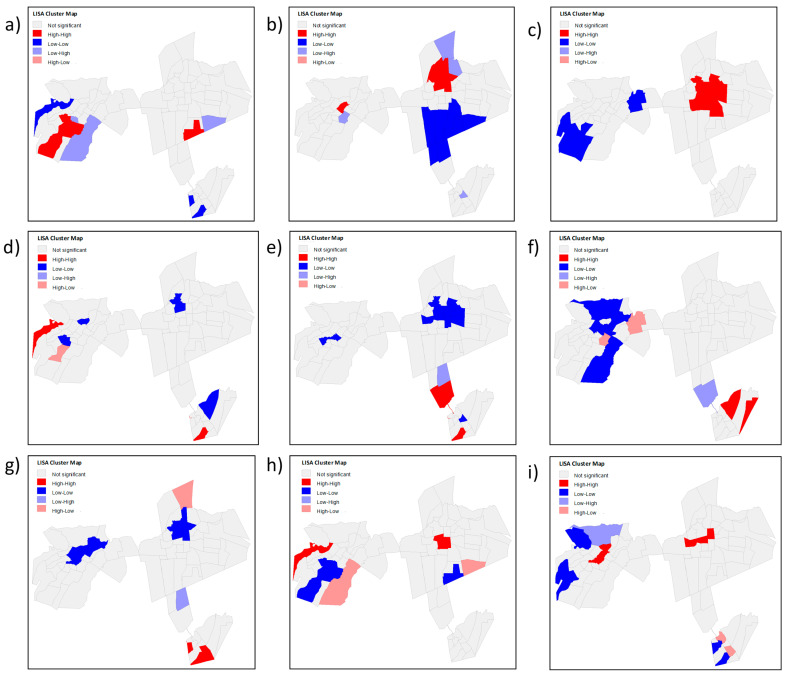
Univariate local autocorrelation maps relating to the total number of COVID-19 cases and the independent variables (PEVI and the indicators that comprise it): (**a**) low income distribution; (**b**) persons aged 60 or over; (**c**) population density; (**d**) people in the situation of poverty and extreme poverty; (**e**) households without water supply and bathroom; (**f**) absence of urban infrastructure; (**g**) household density; (**h**) potential epidemic vulnerability index; (**i**) total number of COVID-19 cases per neighborhood.

In this study, the method for measuring the spatial autocorrelation that best suited the data in this study was the queen-type contiguity matrix, which is one of the most used in this type of analysis. Univariate local autocorrelation maps relating to the total number of COVID-19 cases and the independent variables are described in Figure 4.

The significant spatial clusters of the total COVID-19 cases, shown in Figure 4h, indicate a high–high relationship in the Antônio Vieira, Santa Tereza, Franciscanos, Centro de Crato, and Vila Alta neighborhoods; that is, the number of cases was high with neighbors whose number of cases was also high. Conversely, a low–low relationship was evidenced in the Lameiro, Gizélia Pinheiro (Batateiras), Bela Vista, Alto do Rosário, and Rosário neighborhoods, representing areas that could still be protected by public health measures to prevent the increase in cases within these places since three of them had a high and very high PEVI and two average PEVI.

Figure 4h shows a few transition zones; that is, the zones whose numbers of cases were high with neighbors whose numbers of cases were low (high–Low) or the opposite (low–high), were, respectively, the Cirolândia and Barbalha Center (in the first link) and Parque Recreio.

**Figure 5:**
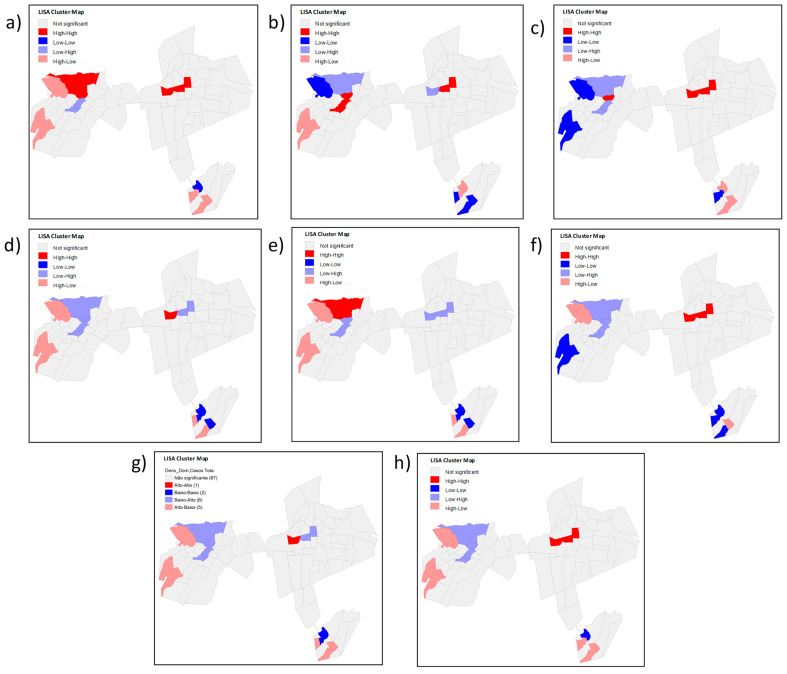
Local bivariate correlation map using the total number of cases of COVID-19 in the neighborhoods as a dependent variable and PEVI and its indicators as independent variables: (**a**) low income distribution; (**b**) persons aged 60 or over; (**c**) population density; (**d**) people in the situation of poverty and extreme poverty; (**e**) households without water supply and bathroom; (**f**) absence of urban infrastructure; (**g**) household density; (**h**) total number of COVID-19 cases per neighborhood.

In the bivariate spatial correlation, in cases of the dependent variable, as shown in Figure 5, three of the five neighborhoods mentioned above show a high–high autocorrelation (Antônio Vieira, Santa Tereza, and Franciscanos) while also showing a statistically significant relationship of the high–high type with independent variables: PEVI, population density, the absence of urban infrastructure and low income distribution, all indicated a high number of confirmed cases with a high independent variable.

Still, in this region, the Antônio Vieira neighborhood presented a low–high relation for the independent variable people aged 60 years or older; that is, a low number of confirmed cases and a high number of older people acted as a transition zone within the Santa Tereza and Santa Tereza and Franciscanos neighborhoods. Conversely, these last neighborhoods showed a high–low relationship for the indicator of people in poverty, extreme poverty, and household density, with transition zones being the first.

Despite not showing clusters that had a positive direct relationship with the PEVI, other areas with a high number of confirmed COVID-19 cases could be justified by the statistically significant relationship between LISA and the indicators that make up the PEVI. The Parque Recreio neighborhood had a very high PEVI (Figure 2) and a low number of confirmed cases (Figure 3), with a bivariate low–high spatial correlation between these variables (Figure 5h) and a positive high–high relationship alongside variables with a low–income distribution (Figure 5c) and the absence of urban infrastructure (Figure 5d). Therefore, it is evident that low income and worse sanitary conditions were related to a high number of confirmed cases of COVID-19.

Conversely, for example, the Gizélia Pinheiro neighborhood (Batateiras) has a high PEVI (Figure 2) and a low number of confirmed cases (Figure 3), with a positive low–low relationship for the independent variables population density and people aged 60 years or older and is located in a neighboring region with a high–low relationship with the PEVI (Parque Recreio).

The spatial correlation in the Lameiro neighborhood presented a negative relationship of the high–low type between the number of cases and PEVI (Figure 4h), meaning that it has a high PEVI and a low number of cases. It may reflect social situations since underreporting is associated with difficulties in accessing health systems.

The presence of spatial clusters formed by the Centro de Barbalha neighborhood was of the low–low type. However, its neighbor with statistical significance presented a high–low relationship; that is, a high PEVI and a low number of cases, which may mean that this contributed to maintaining low cases with each other.

## 4. Discussion

The economic development of the Crajubar urban agglomeration began in the second half of the 20th century and increased in the late 1960s. Since then, this region consolidated its importance in Ceará, influencing the entire southern region of the state and municipalities on the border, while the states of Pernambuco, Paraíba, and Piauí offered products and services. Thus, the need to implement public health planning policies in these interior areas became intensified [30].

The exploratory analysis of spatial data verifies the relationship between geographic entities by the value of a particular indicator or a sample (HDI, precipitation, mineral content in the soil, etc.). It corroborates the existing relationship between neighboring areas. The local spatial association indicator is a statistical parameter that presents values that are proportional to the global statistics so that it can allow the degree of similarity or difference in each event concerning the closest events to be described [31].

In a geostatistical analysis to detect spatiotemporal patterns of the COVID-19 epidemic at the municipal level in Mexico over 12 months, it was found that the pandemic was characterized by parallel epidemics in different groups, but without joint assessments regarding the social vulnerability of that population [32]. A spatial assessment was also key to understanding spatial spread during the early stages of the COVID-19 pandemic in mainland China [33].

It is known that vulnerability aggravates the spread of COVID-19 and is crucial to combating the disease. In this way, its analysis for controlling, preventing, and promoting health at the national and regional levels must be considered a priority [19].

Research in the literature shows that the vaccination in descending order of distribution to the calculated epidemic vulnerability index showed a significant result with an average of 5.0% with fewer cases of infection, 9.4% fewer cases of death, and a 3.5% lower rate of mortality than other routes of vaccine delivery [22].

Although COVID-19 has the potential to impact all social groups, from the most vulnerable to those with a high purchasing power, they are disproportionately affected, especially if assertive health promotion actions are absent. Action plans, transmission reduction, and assistance promotion must be watched from the perspective of equity in health [34].

In 2022, a study took a holistic approach and analyzed the spatiotemporal clustering patterns and sociodemographic determinants of COVID-19 infections in Helsinki, Finland, where it was identified that high–high clusters and relatively high–risk areas emerged mainly in the eastern neighborhoods, which are socioeconomically vulnerable, with some exceptions revealing local outbreaks in other areas. The findings can also be applied to any other global location with similar datasets contributing to existing geospatial knowledge of the COVID-19 pandemic at a local scale [24].

In turn, the cross-sectional study by Abedi, 2020, in the United States evaluated the association between infection and the mortality rate of COVID-19 and demographic, socioeconomic, and mobility variables in 369 municipalities. Of these, those with higher rates of disability and poverty had a higher death rate. The observed inequality could be explained by low access to essential services alongside care and poverty [20].

Another study in Malaysia assessed the spatiotemporal propagation pattern of the COVID-19 pandemic over 13 months since the index case, emphasizing the spatial autocorrelation of high-risk clustering events and the spatial sweep clustering pattern of transmission. In this case, daily local spatial autocorrelation (LISA/local Moran I) was able to measure the dynamics and intensity of the spatial spread of the disease based on the population at risk [35].

In the present study, the Moran index demonstrated the existence of spatial auto-correlation, evidencing spatial dependence between the total number of confirmed cases of COVID-19, the PEVI, and its indices for neighborhoods of Crajubar. Although the global index did not show a statistically significant difference, the local indicator (LISA) allowed clusters of these variables to be verified, mainly in the extreme regions of the center and periphery of Crajubar.

This spatial distribution proved to be heterogeneous, with a disproportionate distribution of the disease between neighborhoods and sometimes with an inverse relation to the PEVI, which may be related to underestimation attributed to a lack of knowledge and difficulties in accessing the health system and diagnostic means. Overall, the number of COVID-19 cases was high in neighborhoods with lower socioeconomic levels, but some areas with high PEVI also had a low number of cases.

This fact indicates the need for deeper local investigations into the causes and consequences of the spatialization of the neighborhoods mentioned above and other micro-regions since the demographic transition implies new demands and social and health needs that require a reorientation of public policies [28].

Population-based ecological analyses are also being applied to the vaccination and spatial distribution of all fully vaccinated children and adolescents and have identified a heterogeneous distribution, with spatial clusters of the lowest vaccination rates occurring mainly in municipalities with marked socioeconomic disparities and social vulnerability [36].

Coping with the COVID-19 pandemic in all regions varies according to social aspects, such as gender, age, education, place of residence, and beliefs. Many factors can affect the speed with which effective disease control practices are implemented, such as information campaigns, local health practices, and social behavior [37].

Several regions of the country, including the northeast, are marked by intra-regional divergence and, in general, territorial heterogeneity associated with sociodemographic variables and the individual characteristics of each population, highlighting the need for micro-region studies and the implementation of health measures [38]. Consequently, this research is helpful as a basis for spatial analyzes of other micro-regions for direct effective measures of health promotion and prevention for COVID-19 and other diseases while implementing interventions to support more vulnerable groups.

In addition, despite the evolution of the Brazilian public health system, it is worth mentioning that neglected diseases or those associated with chronic health care in low–income populations may emerge secondary to COVID-19 and have an even more dismal impact [2].

Therefore, it is crucial to combat the spread of COVID-19 with a multicentric approach based on public health promotion and prevention measures, guided by epidemiological evidence, as well as knowledge of the most vulnerable population groups [39], not only in the areas that express a high number of cases.

## 5. Conclusions

The influence of vulnerability indicators on prevalence showed areas that could be targeted by public policies to impact the occurrence of COVID-19. Intervention programs must be implemented to support the most vulnerable groups; therefore, for an early articulation to face future epidemics and pandemics of infectious and contagious diseases, and in order to guide public policies and local health authorities in the implementation of mitigation strategies, it is essential to understand the benefits of a spatial analysis approach in all parts of the world, associated with social determinants, in micro or macro spatial analysis. Future research should be undertaken using COVID-19 surveillance data in collaboration with local health authorities and should be encouraged to elucidate these complex spatiotemporal and social patterns to direct current and future pandemic mitigation and control efforts.

## Figures and Tables

**Table 1:** Indicators that integrate the Potential Epidemic Vulnerability Index.

Indicators	Description
Income distribution	Average monthly nominal income of people aged ten years or older, including those without income.
People in poverty and extreme poverty	Percentage of people with household income per capita equal to or less than R$ 127.00 per month in August 2010.
Population density	Number of inhabitants per km^2^.
People aged 60 years or older	Percentage of people aged 60 and older.
Households without water supply and toilet	Percentage of households without water supply and bathroom.
Absence of Urban Infrastructure	Percentage of households in areas with open sewage, no public lighting, no paving, and no electricity supply.
Household Density	Ratio between the total population and the number of neighborhood homes

**Table 2:** Description of notified cases of COVID-19 in the urban area.

Variables		Frequency *n* (%)	Mean *	Median *	Standard Deviation *	Min–Max *
COVID-19 cases		62,290 (100)	769.01	658	630.17	2–3721
Race	Asian	4790 (7.6)	59.13	42	50.44	0–187
	White	11,490 (18.4)	141.85	110	131.74	0–606
	Indigenous	82 (0.13)	1.01	0	1.62	0–8
	Black and Brown	45,928 (73.7)	567.01	470	472.51	2–2971
Sex	Women	35,515 (57)	438.45	369	360.93	2–2138
	Men	26,775 (42.9)	330.55	299	270.84	0–1583
Age Range (years)	0–19	8431 (13.5)	104.08	87	84.14	0–445
	20–39	28,104 (45.1)	346.96	305	284.28	0–1727
	40–59	18,553 (29.7)	229.04	194	190.42	2–1148
	60 and older	7202 (11.5)	88.91	64	86.66	0–401

* The variables refer to the analyzes carried out by neighborhood.

## Data Availability

The data supporting this study’s interpretations will be made available by the authors if requested.
